# Zebras, Intransigence & Semantic Apocalypse: Problems for Dispositional Metasemantics

**DOI:** 10.1007/s11406-015-9684-5

**Published:** 2016-02-04

**Authors:** James Andow

**Affiliations:** University of Reading, Reading, UK

## Abstract

Complete information dispositional metasemantics says that our expressions get their meaning in virtue of what our dispositions to apply those terms would be given complete information. The view has recently been advanced and argued to have a number of attractive features. I argue that that it threatens to make the meanings of our words indeterminate and doesn’t do what it was that made a dispositional view attractive in the first place.

## Introduction

How do our terms come to mean what they do? A prima facie plausible idea is that it has something to do with our dispositions to apply those terms. It is attractive to say that what our terms mean has something to do with how *we* are, rather than being due to completely external considerations. A dispositional account promises to do justice to this idea without getting stuck with typical problems faced by traditional internalists—although this is not a point I’ll defend here. Moreover, it can, if desired, capture typical externalist intuitions, e.g., about Twin Earth, without giving up on the idea that what ultimately grounds the meaning of my words concerns facts about my current state.^1^

A straightforward dispositional metasemantic account (SDA), however, doesn’t withstand much scrutiny.

SDA: A linguistic expression E means some object, property, kind, relation, etc., X, in the mouth of speaker S, in virtue of the fact that S would be disposed to apply E to X.

SDA faces two main problems: (1) it is very unclear what enables a single term E to mean the same when I use it as when you use it for we surely have different dispositions to apply pretty much any term if only ever so slightly different; (2) SDA threatens to make it almost impossible to misapply an expression for there’s no obvious sense to the idea that one might have applied an expression to something in certain circumstances despite not being disposed to do so.

Recently a new variety of dispositional metasemantics has been proposed which promises to overcome the problems faced by a more straightforward account such as SDA. The next section outlines the account in question: the complete information dispositional account. Then, in the rest of the paper, I argue that this account faces a number of important objections and consider various ways one might attempt to respond to these objections.^2^ Ultimately, I conclude that the complete information dispositional account threatens to make the meanings of our words indeterminate and loses track of what it was that made a dispositional view attractive in the first place.

## CIDA

What sets a complete information dispositional account (CIDA) apart from SDA? The difference is that, according to CIDA, meaning is grounded in speakers’ dispositions to apply terms given *all relevant information* or *complete information* rather than their actual current dispositions.

CIDA: A linguistic expression E means some object, property, kind, relation, etc., X, in the mouth of speaker S, in virtue of the fact that S would be disposed to apply E to X if S had all the relevant information.^3^

Basically CIDA is as SDA but with the following tweak: the relevant dispositions are the dispositions S would have were they to have *all relevant information* or *complete information*. What is this state of having all relevant information supposed to be? The idea is that the relevant state is one in which there is no piece of information apprisal of which would change S’s dispositions to apply the relevant term.

Before moving on, take note of an important feature of the view. This is a feature which might not be immediately obvious. CIDA accepts that meaning is grounded in speakers’ actual current dispositions. How is this so, given the appeal to the counterfactual situation of having complete information? The reason is that dispositions to apply a term given complete information are ultimately grounded in one’s actual dispositions. The difference is that CIDA doesn’t focus simply on one’s actual dispositions *to apply the relevant terms* (like SDA). To see this point, it helps to recognize a distinction between first and second order dispositions. Individuals have certain dispositions to apply terms, i.e., first-order dispositions. An individual may have second-order dispositions concerning these first-order dispositions. An individual may be disposed to change first-order dispositions in response to certain bits of information and to retain their first-order dispositions in response to all other bits of information. In other words, whether one would be disposed to apply a term given complete information is a function of one’s *actual current* dispositions where this includes both first and second order dispositions (modulo the precise contents of ‘complete information’).

While retaining this feature—accepting that meaning is grounded in speakers’ current dispositions—CIDA seems equipped to deal with the issues faced by a more straightforward dispositional account such as SDA. (1) CIDA has sensible things to say about when two speakers mean the same thing when they use a term. For an expression to mean the same in my mouth as yours is for us both to have second order dispositions such that were we to have complete information we would have the same first order dispositions to apply the expression. We might mean the same despite having different first order dispositions. We might mean the same despite having different second order dispositions (e.g., given different starting points). What unifies the meanings of an expression as used by two individuals is that, given complete information, their first-order dispositions would converge. (2) CIDA has sensible things to say about the possibility of misusing terms. To misapply a term is easy, one’s actual current first order dispositions are likely different from those one would have given complete information, and wherever the two diverge, there is scope for misapplication.

One might worry that employing a *counterfactual* device—such as these very well-informed speakers—would result in a view that fails to do justice to the intuitive motivation for considering any kind of a dispositional account in the first place. The reason a dispositional account seems attractive is that it seems able to marry the intuitive sense that meaning should be grounded in facts about the speaker without getting into the difficulties associated with internalist accounts such as descriptivism. That is to say, the advocate of a dispositional view thinks that it is not even remotely plausible to think that the meaning of expressions as used by an individual is completely divorced from that individual’s current tendencies. However, fortunately, on the face of it CIDA’s use of this counterfactual device manages to do justice to this intuitive motivation. What makes it the case that some of my applications of ‘cow’ are correct whereas others are incorrect? For CIDA the answer concerns facts about me now: facts about my dispositions to respond to further information of which I am not currently aware.

Johnson and Nado (2014) do a good job of extolling the virtues of CIDA in a little more detail. I won’t do anymore to motivate CIDA or dispositional views more generally here. Instead, in the following, I consider a number of potential objections to views like CIDA.

## Zebras

The first potential objection to CIDA concerns whether ‘S’s dispositions to apply E given complete information’ picks out a determinate set of dispositions to apply E. To explore this objection, it is helpful to consider CIDA’s relation to the (in some respects) similar idea of *temporal externalism*. Temporal externalism is the view that ‘the future behavior of an individual or his society can affect the content of his thoughts and utterances’ (see Jackman 1999, 2005). One way Jackman describes temporal externalism is to say that it involves deference to future experts (Jackman 2005). This is a somewhat similar device to the idea of oneself and one’s first order dispositions given complete information; both the future expert and the counterfactual you have the benefit of knowing things which the actual current you does not.

Despite any similarity, the cases Jackman uses to motivate temporal externalism in fact suggest a problem for CIDA. Jackman motivates temporal externalism by appealing to various situations in which current meaning seems to depend on how future dispositions to use particular terms would respond to particular findings. For example,

The term ‘Grant’s zebra’ was introduced around 1820 for a type of zebra native to Kenya. A few years later, the term ‘Chapman’s zebra’ was introduced for a morphologically distinct type of zebra found in present-day Zimbabwe. Later still it was discovered that the two types of zebra interbred near the Zambezi River and that, morphologically, one gradually faded into the other. Grant’s and Chapman’s zebras were thus both taken to be races of the species Equus burchilli. However, while that was how our usage of the term actually developed, it seems likely that if the taxonomists had investigated the area around the Zambezi River before they explored Zimbabwe, they would have “discovered” that Grant’s zebra could be found through most of East Africa, gradually changing into a different subspecies as it drifted south. In this counterfactual scenario, ‘Grant’s zebra’ would have been applied to the entire species, not just the race found in Kenya. (366)

The possibility that future discoveries might affect the content of current thoughts and utterances is something which CIDA is happy to accept. That is not a problem for CIDA. The discovered information is presumably part of the ‘complete information’ which is packed into the idea of ‘dispositions given complete information’. The potential problem is the fact that if one is going to accept that (a) the way one’s first order dispositions would change given apprisal of new information is relevant to the meaning of terms as you currently use them, then it is unclear on what grounds one could resist Jackman’s claim that (b) the discovery of bits of information in *different orders* might affect the meaning of terms as you currently use them. Accepting (b) looks problematic for CIDA, but CIDA is committed to (a).

Here’s one way to put the problem: if Jackman is right about cases like the zebra case, the relevant individuals have no determinate *dispositions given complete information*. So it seems that the notion of *S’s dispositions given complete information* employed by CIDA may not fix a single set of dispositions. There is no obvious reason to think that generally speaking I would be disposed to respond in the same way to receiving complete information in order $\{i_{1},i_{2},i_{3}{\dots } i_{n}\}$ and $\{i_{9},i_{7},i_{3}\dots \}$, for instance. So, the worry might be, CIDA has no obvious way to resist the conclusion that many (perhaps all) of my expressions have indeterminate meanings.^4^

I think the most plausible way for the proponent of CIDA to respond starts with noting that the way I have expressed the problem might be thought unhelpful. Why? Because it misses out on the fact that second order dispositions and the order of learning information interact in order to *change* first order dispositions. Why is this relevant? Because an individual *given complete information* is by definition in possession of all information which would lead to a change in their first order dispositions. What the zebra case seems to suggest to me is that this information at least potentially includes information about one’s second order dispositions and one’s counterfactual selves. It does seem plausible, for example, that giving an individual a chance to reflect on the ways that the order in which information is learned might influence their first order dispositions, and this might in turn influence their first order dispositions. For instance, the proponent of CIDA might try to say that on reflection the *actual* taxonomists would recognize that ‘Chapman’s’ and ‘Grant’s’ don’t really pick out genuine groups and revise their taxonomy accordingly.^5^ Nonetheless, it seems to me a little optimistic for the proponent of CIDA to think that in the closest possible worlds in which the actual and counterfactual taxonomists are apprised of each others’ paths of exploration and the effects on each others’ use of terms, the two groups of taxonomists would converge in their first order dispositions.^6^

## Intransigence

The second potential objection is somewhat similar. There are some other reasons to think that we shouldn’t necessarily expect convergence given *complete* information—even if CIDA can say something sensible about cases like Jackman uses to motivate temporal externalism—and that CIDA might thus have the result that many or all of our terms have indeterminate meanings.

The way we have been understanding ‘all relevant information’ or ‘complete information’ is that one is in the relevant state with respect to an expression if there is no new information which would change one’s dispositions to apply the expression. Here’s another way to say the same thing. To have complete information is to have *intransigent dispositions*. This is really just a relabeling of the notion of ‘complete information’ being used. However, the relabeling is useful as it draws attention to a feature of CIDA which one might not otherwise see. The idea of ‘complete information’ might conjure the picture of some single ideal epistemic state. But that isn’t quite right. We can see this by noting that there might be multiple ways in which an individual might come to have the intransigent dispositions which characterize the state of having ‘complete information’.

First, take a toy example. Take someone who uses the word ‘God’ and who applies ‘God’ to the sun (and only the sun) due to some particular religious beliefs. Due to other features of their overall belief set, their faith might be completely insensitive to countervailing evidence, i.e., there might be no evidence or argument which could possibly alter their dispositions to apply ‘God’. They have intransigent dispositions, but we might not automatically have recognized them as appropriate recipients of the label ‘in possession of all relevant information’.

Now, note that there are other, slightly less eccentric individuals who are in very a similar position. For instance, there might be an individual who is not currently in an intransigent position, but for whom certain bits of information would soon place them in one, e.g., the information that leaders in her religion believe that everyone else in the world is deliberately trying to mislead followers of the ‘true faith’. For these slightly less eccentric individuals, note, there might be *multiple* stopping points open to them—multiple intransigent positions in which they could find themselves. They are not doomed to be intransigent appliers of ‘God’ to the sun, apprised of different information they could escape this fate, meaning that their *dispositions given complete information*, viz., first order dispositions *once no further piece of information would change their first order dispositions*, would be rather different.

The take-home message here is that one’s current first order dispositions and second order dispositions are not guaranteed to fix a single set of *first order dispositions given complete information*, because even for a particular individual, term, and time, there can be more than one set of contents assigned to ‘complete information’. This means that it is not guaranteed that our terms have determinate meanings. It is important to note that this is a different worry than that considered in the previous section. What threatens the determinacy of the meaning of our terms is not that what dispositions we have given complete information might be different depending on the order in which we received information. Rather, the threat concerns the possibility that there is more than one set of information which would count as ‘complete’ for a single individual, term and time. The potential sunworshipper’s future doesn’t go one way or the other depending on in which order they become aware of some single set of facts known as ‘complete information’. Rather, their future goes one way or the other depending on which version of ‘complete information’ they receive. The notion of ‘complete information’ or ‘all relevant information’ which CIDA invokes isn’t quite what it seems: it doesn’t obviously always pick out a single epistemic position even given a particular individual and their use of a particular term. ‘Complete information’ or ‘all relevant information’ just picks out any informational state which will render an individual intransigent in their dispositions.

I suspect the proponent of CIDA will want to respond to what I have said so far along the following lines. Why can’t we treat the case of the potential sunworshipper and similar cases as exceptions? They might provide a reason to think that the majority of us are not in a similar situation. However, it is far from clear what principled grounds there might be for assuming that for most language users and most of their terms there is only a single set of true propositions which would constitute ‘complete information’ and render them intransigent in their dispositions. Unless there is some good reason to make this assumption it is on the cards that most of our terms have indeterminate meanings (even setting aside the earlier issues about the order in which information is presented). There may be principled considerations to which the proponent of CIDA can appeal to at this point. However, until they do so, there is a significant theoretical cost to be associated with their position.

How might the proponent of CIDA deal with this potential objection? (1) One natural approach might be to invoke a different notion of ‘complete information’ or ‘all relevant information’ which can’t be captured simply in terms of a state in which one’s first order dispositions are intransigent and which is decoupled from the idea that relevant information is that information apprisal of which would lead to alterations in your first order dispositions. But it seems that unless you also decouple meaning from those aspects of us which mean that there are multiple intransigent positions open to us (and, e.g., give a dispositional account in terms of some idealized agent with perfect information, perfect rationality, etc.) then it won’t solve the problem. Moreover, once this decoupling takes place it is unclear in what sense CIDA has any of the intuitive appeal dispositional views seemed to have, i.e., grounding fact about the meaning of our words in facts about our actual tendencies. (2) Another approach might be to offer something similar to the response I considered in the previous section. This might go as follows: all information which would change one’s dispositions is relevant information; in these supposedly intransigent cases, the individual’s dispositions are not really intransigent; upon consideration that there were multiple other paths available to them (in some sense) which would also have resulted in different (but also seemingly intransigent) positions, their first order dispositions would change. This is a possible view. However, it remains to be seen why, in principle, we should expect there to be only one intransigent position open to individuals (at least in the vast majority of cases).

## Semantic Apocalypse

Now for a slightly different worry. Above I said that you don’t want an account of meaning which divorces the meaning of an individual’s term from that individual’s current state. One might worry that CIDA falls foul of this desideratum, despite the obvious sense in which CIDA grounds meaning in an individual’s current state.

This worry invokes the possibility of *semantic apocalypse*. Given complete information, it is at least clear that *some* expressions will be abandoned. Take ‘phlogiston’. Given the information we have, we take it to be non-referring. We have not abandoned it in the sense that many of us competently recognize what the world would have to be like for it to apply and fail to apply it. Nonetheless, it *has* been abandoned in the sense that new terms now have to be deployed in order to describe and explain those phenomena in which ‘phlogiston’ was supposed to play a part.

The possibility of semantic apocalypse is the possibility that all or the vast majority of our current vocabulary resembles the case of ‘phlogiston’. Clearly this is a possibility. Unfortunately, it seems things are worse than that. Indeed, once we consider the fact that CIDA invokes the notion of ‘complete information’, semantic apocalypse seems somewhat probable. Consider that complete information is potentially a lot of information. One doesn’t have complete information until there is no fact (of which you remain unaware) apprisal of which would lead to any alteration in one’s first order dispositions. One’s view of the world in such a state seems likely to be very different from that we now have. Indeed, once given access to complete information—viz., every single fact about reality which would change the way in which we talk about it—it seems pretty likely that our entire conceptual framework would be overhauled to the extent that the vast majority of expressions in our current vocabulary would be abandoned or completely revised from the ground up.^7^ It seems pretty likely that there is information out there which would radically restructure the nature of human existence, make us abandon ways of life, abandon technologies, reconsider our values and place in nature, information which would lead us to restructure the political organization of our species, reconsider national boundaries, and the ‘artificial divisions’ which having distinct languages impose upon us. The likely effect of complete information is semantic apocalypse. (Just to be clear – my claim here is not that it is likely we will actually undergo such a shift. Who is to say what volume of information humankind will become aware of before extinction? Rather, the claim is that the probable result of being exposed to all information which would alter one’s dispositions, i.e., complete information, would involve a radical overhaul in semantic dispositions.)

Suppose I’m right that the likely effect of complete information is semantic apocalypse. The implications of CIDA seem somewhat drastic. Our current vocabulary would consist (almost entirely) of words whose meaning is grounded by the dispositions not to use the words to refer to anything; the apocalypse seems to simply decimate our vocabulary—or, at least, that’s a melodramatic way to put it. Of course, the proponent of CIDA can come back and tell us that this isn’t how dispositions work. Given full information, they might point out, one would retain dispositions to apply the expressions (albeit dispositions to apply the terms only in circumstances which will never obtain). However, this retort misses the main point I want to make. The main point I want to make is not that the likelihood of semantic apocalypse renders all our words meaningless or anything like that. The main point I want to make is that, given the likelihood of semantic apocalypse under complete information, CIDA seems to open up a huge undesirable gap between the meaning of our words and our current state. Our meaning no longer seems to retain any connection to our current state in any important sense. Of course, it is true that what makes our current expressions empty is the fact that we have dispositions such that given complete information we would abandon all our current vocabulary. However, I take it that this connection is not sufficient to appease the intuition that any sensible story about meaning needs to retain an important connection between the meaning of words as used by S and the current state of S.

## Conclusion

Complete information dispositional metasemantics may have some promising features. However, for the moment, it also has some worrying features. A plausible defence of a complete information metasemantic account needs to address two important worries which my discussion has highlighted. I do not assume the worries I have highlighted are insurmountable or ultimately decisive. However, they do pinpoint two important theoretical costs which should be borne in mind when assessing the merits of accounts such as CIDA. The two worries I highlighted are as follows. First, there seems to be no in principle reason to think that ‘S’s dispositions to apply E given complete information’ picks out a single determinate set of dispositions in the majority of cases. The proponent of CIDA needs either to bite the bullet and accept that most of our terms, given CIDA, have indeterminate meanings or else provide some such principled reason. Second, my discussion of semantic apocalypse seems to suggest that there could well be *quite generally* a huge gap between our current tendencies in using words and the meaning of our words (not just in specific instances). This seems contrary to the intuitions which provide the motivation for considering dispositional metasemantics in the first place. So, the proponent of complete information dispositional metasemantics needs to either (a) demonstrate, in light of the possibility of semantic apocalypse, that CIDA can nevertheless provide the relevant intuitive connection between our actual current states and our meanings, or (b) demonstrate that CIDA is still an attractive view despite its inability to provide such a connection.

## Footnotes

1 See Johnson and Nado (2014) for details.

2 Some of these ideas elaborate on my remarks in Andow (2015) (a critical review of Booth and Rowbottom 2014).

3 This articulation is from Johnson and Nado (2014).

4 It is, of course, *possible* for the proponent of CIDA to bite the bullet here and accept that most of our language might have indeterminate meaning and not just the bits we might have suspected, like obvious instances of vagueness. My main point is simply to note that there is a large, although not by itself decisive, theoretical cost to be associated with CIDA unless its proponent can provide an in principle reason to think that these sorts of consideration do not apply in the case of most of our vocabulary.

5 Many revisions in zebra taxonomy have in fact been made since the zebra case entered philosophical discussion, see Groves and Bell (2004). Although, these changes have typically not been on the basis of counterfactual speculation (as far as I can make out).

6 Note that if they were to take this route then they couldn’t accept Jackman’s story about the meaning of the actual and counterfactual taxonomists’ terminology. CIDA, as I have presented it, is not compatible with temporal externalism. However, since, as Jackman admits, the general response to temporal externalism is an incredulous stare, I take it that this is no huge theoretical burden for the proponent of CIDA to bear.

7 For those familiar, the most dramatic case of this resembles something like Douglas Adams’ ‘Total Perspective Vortex’ from *The Hitchhikers’ Guide to the Galaxy*.
